# Supplementary material for: Blood Pressure Levels and Triglyceride–Glucose Index: A Cross-Sectional Study from a Nationwide Screening in Mongolia
Source: J Clin Med. 2025 Sep 29;14(19):6890. doi: 10.3390/jcm14196890 (PMC12525516; doi:10.3390/jcm14196890)
Supplement: Supplementary file 1 [file jcm-14-06890-s001.zip › jcm-3864022-supplementary.pdf]

Supplementary Material.

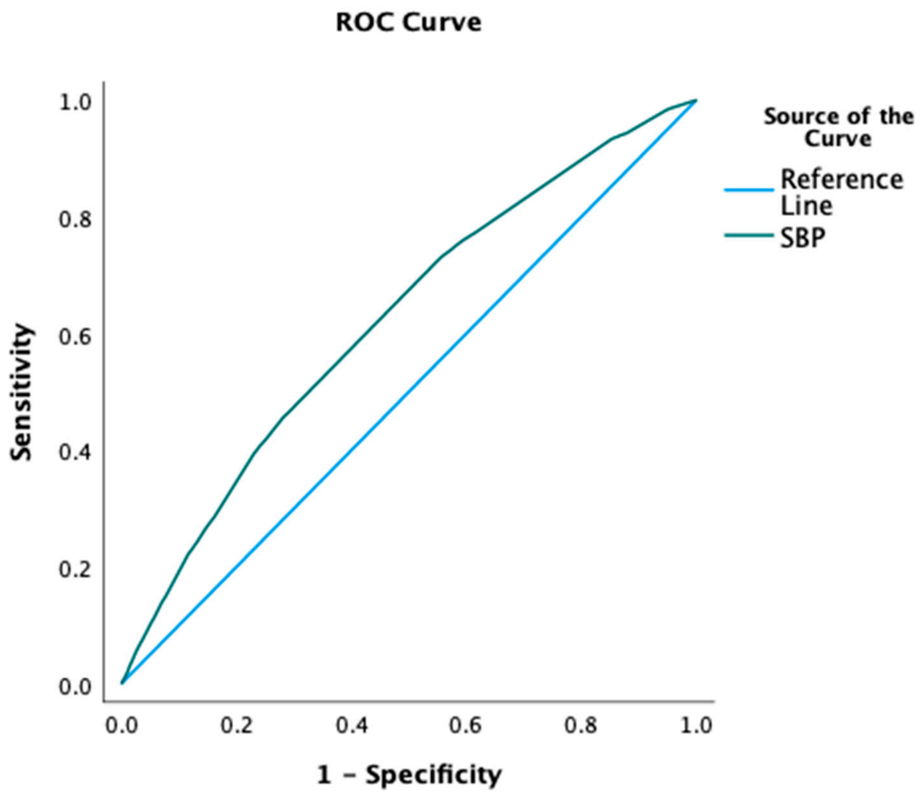

Supplementary Figure S1. ROC curve of systolic blood pressure for elevated TyG index.
